# Supplementary material for: The impact of daily caffeine intake on nighttime sleep in young adult men
Source: Sci Rep. 2021 Feb 25;11:4668. doi: 10.1038/s41598-021-84088-x (PMC7907384; doi:10.1038/s41598-021-84088-x)
Supplement: Supplementary file 1 — Supplementary Information [file 41598_2021_84088_MOESM1_ESM.pdf]

Supplemental Material

The impact of daily caffeine intake on nighttime sleep in young adult men

Janine Weibel[1,2], Yu-Shiuan Lin[1,2,3], Hans-Peter Landolt[4,5], Joshua Kistler[1,2], Sophia Rehm[6], Katharina M. Rentsch[6], Helen Slawik[7], Stefan Borgwardt[3], Christian Cajochen[1,2]*, Carolin F. Reichert[1,2]*

[1] Centre for Chronobiology, Psychiatric Hospital of the University of Basel, Basel, Switzerland
[2] Transfaculty Research Platform Molecular and Cognitive Neurosciences, University of Basel, Basel, Switzerland
[3] Neuropsychiatry and Brain Imaging, Psychiatric Hospital of the University of Basel, Basel, Switzerland
[4] Institute of Pharmacology and Toxicology, University of Zürich, Zürich, Switzerland
[5] Sleep & Health Zürich, University Center of Competence, University of Zürich, Zürich, Switzerland
[6] Laboratory Medicine, University Hospital Basel, Basel, Switzerland
[7] Clinical Sleep Laboratory, Psychiatric Hospital of the University of Basel, Basel, Switzerland
*Authors contributed equally to this work

Table 1. Sleep-wake times derived from the subjective sleep logs prior the in-lab part of the study.

Parameter	Placebo	Caffeine	Withdrawal	Condition Effect
Bedtime	23.15 ± 0.13	23.32 ± 0.15*	23.27 ± 0.14	$F_{2,37}$ = 4.59; p = 0.02
Lightsoff	23.24 ± 0.15	23.41 ± 0.16*	23.37 ± 0.14	$F_{2,37}$ = 3.99; p = 0.03
Lightson	7.31 ± 0.16	7.46 ± 0.19	7.45 ± 0.16	$F_{2,33.1}$ = 3.12; p = 0.06
Wake-up	7.10 ± 0.15	7.24 ± 0.15*	7.26 ± 0.15	$F_{2,36.9}$ = 3.56; p = 0.04
Time in bed	8.09 ± 0.07	8.04 ± 0.08	8.08 ± 0.06	$F_{2,35.1}$ = 0.14; p > 0.8

Represented are means ± standard errors. Mixed model analysis of variance was performed (PROC MIXED) using the statistical software SAS (SAS Institute, Cary, NC, USA; version 9.4). Contrasts were calculated by the LSMEANS statement and degrees of freedom were adjusted based on Kenward and Roger[1].
*p < 0.05 compared to the placebo condition after correction for multiple comparisons.

Table 2. Sleep-wake times estimated from actimetric data.

Parameter	Placebo	Caffeine	Withdrawal	Condition Effect
Sleep start	23.40 ± 0.18	23.82 ± 0.17*	23.83 ± 0.15*	$F_{2,26}$ = 6.09; p < 0.01
Sleep end	7.08 ± 0.20	7.44 ± 0.19*	7.41 ± 1.16*	$F_{2,25.6}$ = 4.39; p = 0.02
Assumed sleep	7.60 ± 0.09	7.52 ± 0.12	7.52 ± 0.13	$F_{2,31.6}$ = 0.16; p > 0.8

Represented are means ± standard errors. Mixed model analysis of variance was performed (PROC MIXED) using the statistical software SAS (SAS Institute, Cary, NC, USA; version 9.4). Contrasts were calculated by the LSMEANS statement and degrees of freedom were adjusted based on Kenward and Roger[1]. Thirteen datasets were excluded from the analyses due to technical problems.
*p < 0.05 compared to the placebo condition after correction for multiple comparisons.

References

1 Kenward, M. G. & Roger, J. H. Small sample inference for fixed effects from restricted maximum likelihood. *Biometrics* **53**, 983-997, doi:10.2307/2533558 (1997).
